# Point-Counterpoint: Should MRSA nares testing be used as a tool for vancomycin de-escalation outside of pneumonia?

**DOI:** 10.1128/jcm.01309-25

**Published:** 2026-04-21

**Authors:** Dan Ilges, Drew T. Dickinson, Jennifer M. Bosquez, Erin H. Graf

**Affiliations:** 1 Department of Pharmacy, Mayo Clinic 23386 , Phoenix, Arizona, USA; 2 Northwell Health Clinical Laboratories, New York, New York, USA; 3 Department of Laboratory Medicine and Pathology, Mayo Clinic Hospital-Phoenix 23386 , Phoenix, Arizona, USA; Vanderbilt University Medical Center, Nashville, Tennessee, USA

**Keywords:** MRSA nares, MRSA nasal, antimicrobial stewardship, de-escalation, antibiotic de-escalation, pneumonia, diagnostic stewardship

## Abstract

Vancomycin is broadly used to treat gram-positive infections in both inpatient and outpatient settings. Its use is primarily for the treatment of methicillin-resistant *Staphylococcus aureus* (MRSA), which is a common cause of pneumonia, skin and soft tissue infections, and bloodstream infections. Vancomycin therapy requires close clinical monitoring to maintain therapeutic efficacy and, importantly, patient safety. Vancomycin use is associated with acute kidney injury in up to 20% of cases, a number that can be driven down by pharmacokinetic dosing protocols. However, the best mitigation against adverse outcomes associated with vancomycin is to ensure it is only used when needed and rapidly de-escalated when not. As such, many stewardship programs, who are tasked with the safe and effective use of antimicrobial agents, have focused on optimizing vancomycin use. There is a strong correlation with MRSA colonization and infection, and a negative MRSA nares screen has a good negative predictive value for MRSA pneumonia (although this association is less well documented for other infections). As such, detection of MRSA in nares swabs, by nucleic acid amplification tests (NAATs) or culture, is a logical tool for stewarding vancomycin. However, the application of MRSA screening tests as MRSA diagnostics is complex, as these tests are cleared by the U.S. Food and Drug Administration for the detection of MRSA colonization, and not for therapeutic decision making. In this issue of the Journal of Clinical Microbiology, the issue of MRSA nares tests is debated by a clinical pharmacy team and the laboratory. The authors review the clinical evidence and regulatory background in which an MRSA nares NAAT may be used to inform treatment decisions.

## POINT

### Following the nose: the case for expanding off-label methicillin-resistant *Staphylococcus aureus* nares screening

Vancomycin was approved for medical use in the United States in 1958 and remains on the World Health Organization’s List of Essential Medicines. Despite the availability of newer antibiotics with activity against methicillin-resistant *Staphylococcus aureus* (MRSA) (e.g., daptomycin, linezolid, tedizolid, telavancin, ceftaroline, and ceftobiprole), vancomycin remains the most used anti-MRSA antibiotic by far and often represents the most used antibiotic overall in hospital settings (1).

Despite the frequency of intravenous vancomycin use, this agent is associated with considerable risks. Originally nicknamed “Mississippi mud” due to impurities, vancomycin was initially associated with high rates of acute kidney injury (AKI) (2). Nowadays, vancomycin has fewer impurities and can be dosed more precisely to avoid the nephrotoxicity associated with supratherapeutic exposure (e.g., lower trough goals or area under the curve:MIC-based dosing) (3). Despite these advancements, patients receiving intravenous vancomycin can still experience AKI at rates near 20% (4). The risk of AKI with vancomycin increases when combined with other nephrotoxins frequently encountered in the hospital (e.g., contrast for imaging) or when the need for dose adjustment is not recognized promptly (e.g., rapidly changing renal function in critically ill patients). AKI is associated with longer hospitalizations, higher costs, and increased mortality (5, 6). Apart from AKI, vancomycin is also notorious for causing infusion reactions and contributing to a large portion of hospital medication errors (7–9).

**Fig 1:**
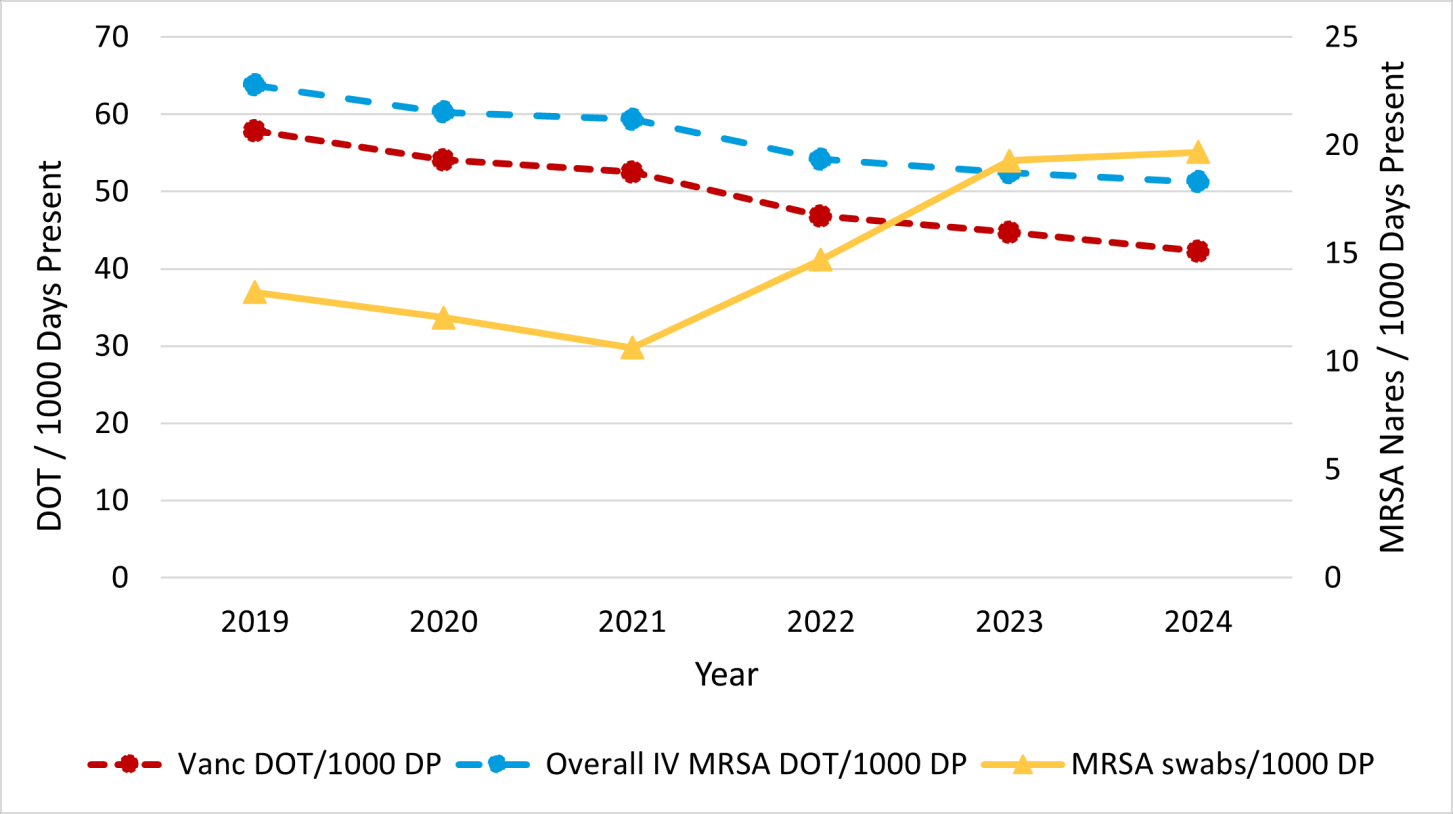
Anti-MRSA antibiotic vs MRSA swab utilization per 1,000 days present over time at Mayo Clinic Arizona. DOT, days of therapy; DP, days present.

Screening for MRSA colonization in the nares, usually via nucleic acid amplification test (NAAT), is one tool aptly equipped to help reduce the use of toxic anti-MRSA treatments, including for indications outside of pneumonia (10, 11). Patients with negative MRSA NAAT performed on a nasal swab (herein referred to as an “MRSA swab”) have been shown to receive less vancomycin and be at lower risk of AKI compared to patients with positive MRSA swabs (12). At our institution, intravenous vancomycin use as measured by days of therapy/1,000 days present (DP) has been steadily downtrending, from 57.2 in 2019 to 38.7 in 2024, which is inversely correlated with the number of MRSA swabs/1,000 DP (Pearson correlation *r* = −0.822, *P* = 0.045, for all anti-MRSA agents, and *r* = −0.815, *P* = 0.048, for IV vancomycin alone; Fig. 1).

Studies show negative MRSA swabs clearly save patients from IV vancomycin administration (and by association prevent vancomycin-associated AKI), but are they *appropriate* to guide vancomycin discontinuation? The use of MRSA swabs for de-escalation in pneumonia has been well established (13). There is biological plausibility in predicting lower respiratory disease with upper respiratory colonization. However, the use of MRSA swabs to rule out MRSA infections at more distal body sites is subject to controversy. Nevertheless, data are encouraging that anti-MRSA agents can be safely stopped for a variety of infectious syndromes when an MRSA swab is negative.

The largest cohort demonstrating the utility of MRSA swabs in de-escalation comes out of the Department of Veterans Affairs (14). Mergenhagen and colleagues evaluated the concordance of MRSA swabs collected within 7 days of a clinical culture among 245,833 adult inpatients over 11 years. The authors found a negative MRSA swab had a 98.6% negative predictive value (NPV) for MRSA detection in intra-abdominal cultures, 98.5% NPV in central nervous system cultures, 96.5% NPV in blood cultures, and 93.1% NPV in wound cultures (14). Smaller studies have confirmed these results. MRSA swabs also demonstrate very high NPVs for bloodstream infections, upwards of 99% (15, 16). In studies of critically ill patients with intra-abdominal infections, negative MRSA swab NPVs range from 91% to 99% (17–19). A systematic review and meta-analysis of MRSA swabs used in diabetic foot infection (DFI) found NPVs ranging from 78% to 94% across six studies (20). More recent data are varied, with NPVs in DFIs ranging from 69% to 94% (12, 21, 22). MRSA nasal swabs in the setting of osteomyelitis have demonstrated similar high utility to DFI with contemporary NPVs estimated around 95% (23). Compared to other infections, the certainty in de-escalating anti-MRSA therapy in skin and soft tissue infections (SSTIs) is lower. NPVs for purulent SSTIs are estimated between 82% and 88%, 80% for non-purulent SSTIs, and 73%–81% for all SSTIs (11, 24).

We must acknowledge that while NPVs for MRSA detection in many syndromes are above 90%, the NPV of MRSA swabs is highly influenced by the prevalence of MRSA infections (25). In the above studies, the prevalence of MRSA infections was regularly below 25%. With a low pre-test probability, it is reasonable to ask whether MRSA swabs should be collected in the first place. However, evaluating the risk of an MRSA infection is difficult, and clinicians are not well equipped to do so based solely on pre-test probability. In a study evaluating MRSA SSTI risk factors in the emergency department, a negative MRSA swab had a greater NPV for MRSA infection compared to all items on a survey designed to evaluate MRSA infection risk. Prescription of an MRSA-active agent also had a lower NPV compared to MRSA swab, suggesting MRSA swabs outperform clinician judgment (26).

Similarly, is the risk of false-positive MRSA swab results, particularly in low-prevalence settings. In very low-prevalence infection sites, such as intra-abdominal infections, most positive MRSA swabs are expected to represent false-positive results (17). We contest that the test may have low utility in diagnosis and de-escalation in these instances. However, infections such as purulent SSTIs can have MRSA prevalence as high as one-third of patients, increasing the probability of true MRSA infection to greater than 50% (24). As such, SSTIs may be one of the more useful realms outside of pneumonia for MRSA swab screening.

One final consideration is whether misinterpretation of MRSA swabs leads to inappropriate de-escalation when falsely negative and thus patient harm. We searched PubMed using the query (MRSA nares) AND ((false negative) OR (inappropriate de-escalation)), which returned 10 results, none of which suggested harm from antibiotic de-escalation in response to falsely negative MRSA swab results. Thus, to the best of our knowledge, harm associated with this practice has not been demonstrated.

We agree that there is likely misinterpretation of MRSA swabs in clinical practice, particularly where the binary result (positive vs negative) is overinterpreted without more nuanced consideration of MRSA risk factors and local prevalence. For example, a patient with concern for prosthetic joint infection following total knee arthroplasty ought to be treated empirically for MRSA, regardless of the result of a MRSA nares, given the high pre-test probability and the potential for introducing MRSA into the joint from the operating room (27). However, we also believe that the potential benefits of stopping unnecessary, error-prone, and toxic vancomycin provide a compelling argument for expanding the use of MRSA swabs outside of pneumonia. Put into perspective, the use of MRSA swabs to facilitate vancomycin cessation is akin to selecting a narrower spectrum beta-lactam, such as ceftriaxone, for a patient with Enterobacterales bacteremia without *bla_CTX-M_* detection by rapid PCR. In both instances, the risk of using narrower therapy and missing the culprit pathogen is non-zero. However, in both instances, the information generated from the test (whether positive or negative) reinforces the pre-test probability and influences the post-test probability for an individual patient, providing additional information. Reassuringly, *S. aureus* readily grows in microbiological cultures, even with pre-treatment, adding an additional backstop in rare instances of discordance between nares screening and clinical cultures (28).

Ultimately, our responsibility in healthcare is to put the needs of the patient first. Viewing this from the perspective of avoiding harm, liberalizing off-label utilization of MRSA swabs to adjudicate risk of MRSA infection and prevent unnecessary administration of toxic antimicrobials seems permissible, particularly when weighed against the counterpoint that regulatory requirements should limit application of the test. However, interpretation of the test should always be in the context of the clinical situation. In situations where the MRSA swab is negative but the patient is critically ill and/or the syndrome/history suggests a heightened risk of MRSA, we advocate for relying on clinical judgment rather than anchoring on the result of the nares screening.

**Dan Ilges and Drew T. Dickinson**

## COUNTERPOINT

### Use of MRSA nares colonization screening for vancomycin deescalation, outside of pneumonia, results in inappropriately testing for a result already known to stop a medication the patient did not need

#### Is turning a test into a laboratory-developed test (LDT) “ok” with the FDA when the lab does not know it is an LDT?

Over the last two decades, the laboratory community has seen a welcome replacement of labor-intensive and slow culture-based methods with rapid, FDA-cleared molecular testing. Although these advancements are tremendously beneficial to patient care, the speed and ease of rapid molecular testing have, in some instances, contributed to over-ordering and unintended consequences. Before FDA-cleared molecular assays became available, MRSA nares colonization screening for infection control purposes primarily used selective agars that took 1–3 days of incubation and workup to determine colonization status. As a result, nares culture screening was not timely enough to guide potential changes in anti-MRSA therapy, since relevant source-related cultures (i.e., respiratory, wound) are typically finalized within a similar time frame. As of 2025, there are at least seven FDA-cleared molecular assays for the detection of MRSA in nasal swabs (29). All seven clearly state in the instructions for use that the test is “not intended to guide treatment.” Not long after FDA clearance of molecular assays, numerous publications emerged showing reasonably high sensitivity of nares screening for the prediction of non-MRSA pneumonia, guiding subsequent de-escalation of vancomycin upon a negative test (30, 31). Importantly, this is considered off-label use of a test, and there are now at least two FDA-cleared options for the detection of MRSA in lower respiratory tract samples intended to guide treatment. Nonetheless, there are some cases in which sputum is not obtainable, despite a diagnosis of pneumonia, and the value of MRSA nares screening may be important, given the reasonably high sensitivity, suggesting a negative result is reliable for de-escalation of anti-MRSA therapy.

While MRSA colonization is well described as a risk factor, it is not required for development of MRSA-associated disease (32). From a microbiological and anatomical standpoint, colonization of the nasal passages can plausibly result in spread via direct communication into the lower respiratory tract. In contrast, it is less clear how nasal colonization can predispose one to MRSA infections outside of the respiratory tract, such as SSTIs, osteomyelitis, endocarditis, and sepsis. In the last few years, groups have explored the use of MRSA nares screening to predict lack of MRSA involvement in non-pneumonia infections. Again, this is an off-label use of a test, which in theory should require the laboratory to perform an LDT validation study for each specific indication. However, since the sample type, in this case a nasal swab, is the same, laboratories may be unaware of the intended off-label use. This distinction of off-label use as an LDT is particularly important, given that in May 2024, the FDA published a final rule requiring laboratories that perform LDTs to comply with standards akin to commercial manufacturers (33). These standards would have been beyond the financial and staffing capabilities of the vast majority of clinical laboratories and would have resulted in the elimination of many LDTs, including off-label use of FDA-cleared tests. The premise of the final rule was that LDTs, including off-label use of FDA-cleared tests, can and have caused patient harm and therefore require clearance or approval for intended use under the FDA’s expertise. Due to the agency of the Association for Molecular Pathology and American Clinical Laboratories Associations’ respective lawsuits, that final rule was vacated (34). However, after repeated threats and attempts at LDT regulation by the FDA, it is likely that this issue is not over. It is worth noting that, in the final rule, reference to the Food, Drug, and Cosmetic Act was made to allow off-label use of FDA-cleared testing at the physician’s discretion, akin to off-label use of antimicrobials, under the “practice of medicine” on an individual patient evaluation basis. However, routine and typically protocolized off-label use of MRSA nares screening for treatment modifications would not fall under this carve-out. Thus, the burden would fall on the laboratory to fully validate and comply with reporting requirements for off-label MRSA nares screening.

### Sensitivity, not negative predictive value, should be emphasized

In an ideal world, all testing would have high clinical sensitivity and specificity for a particular disease; however, this is often not the case for a variety of reasons that are beyond the scope of this counterpoint. As a primer, sensitivity is the ability of a test to correctly identify true positives, while specificity is the converse. These calculations are not biased by disease prevalence. On the other hand, predictive values are probabilistic calculations and heavily impacted by the presence or absence of the target or disease in the population studied. When using a test for “rule-out” purposes, high sensitivity becomes the most important factor to ensure a true case is not missed and therefore inadequately treated, leading to potential morbidity and mortality. Beyond pneumonia, MRSA nares colonization screening has been studied for MRSA-related infection rule out in a variety of clinical syndromes, including sepsis/bacteremia, septic arthritis, and osteomyelitis, all with poor sensitivity. In the case of MRSA nares inference to “rule out” MRSA as the etiology of SSTIs, which is the most commonly studied clinical syndrome and will be the focused example in the remaining paragraphs, the sensitivity is close to the flip of a coin, reproduced across numerous studies (Table 1). Essentially, all studies promoting the benefit of MRSA nares colonization screening for empiric treatment modifications in non-pneumonia infections cite the high NPV, but that argument is fatally flawed by the bias of very low prevalence. Take the Noeldner et al. three-center study, which included admitted patients with SSTI and found an 11.7% MRSA colonization prevalence via nares swab PCR with only 20 direct wound/tissue cultures positive for MRSA in a 1-year period, of which 9 had negative MRSA nares screens (sensitivity of 55%) (16). Because of the large number of cases with MRSA-negative wound/tissue cultures, the NPV is pushed up to 92.7%. Similarly, Mergenhagen et al. published one of the most cited studies in this area, pulling findings from all Veterans Affairs medical centers across a 10-year time frame (14). For SSTI, they did not break out the overall prevalence of MRSA-positive wound/tissue cultures in this cohort, but it was 8.1% for all suspected source of infection cultures in the study (including respiratory). In the cases with MRSA-positive wound/tissue-associated cultures, 54,073 of the 136,078 tested negative for MRSA nares colonization (59.8% sensitivity). Despite the implications that nearly half of the just over 100,000 patients with true MRSA SSTI who needed anti-MRSA therapy would be inappropriately narrowed, the team still concluded the high NPV of 93.1% justifies use of MRSA nares colonization testing for empiric therapy modification. Contrast these findings with a study from Acquisto et al. performed in a large emergency department in upstate New York of patients presenting with SSTI. In this cohort, the prevalence of MRSA SSTI was 44.8%. However, only 30 of the 52 cases with culture-proven MRSA SSTI had a positive MRSA nares screening test result (57.6% sensitivity) (26). As expected, due to the higher MRSA prevalence, the NPV was low at 72.8%. Predictive value is useful when asking the question “if the test is negative, how likely is it that my patient does not have the disease?” When that predictive value of the test is nearly equivalent to the pre-test probability that the patient does not have an MRSA-associated SSTI, it suggests the test is not necessary. While the cohorts are generally much smaller, similar evidence of poor sensitivity of MRSA nares colonization screening for prediction of non-MRSA infection has been demonstrated in other non-pneumonia processes, including sepsis, septic arthritis, and osteomyelitis (14, 16, 23).

**TABLE 1:** Studies evaluating the accuracy of MRSA nares molecular colonization testing to predict MRSA-associated SSTI

Reference	Prevalence of MRSA (no. with positive MRSA in culture/no. of total cultures)	Sensitivity of MRSA nares molecular screening (no. of positive molecular results/no. of cases with MRSA-positive cultures)
Noeldner et al. (16)	11.7% (20/171)	55% (11/20)
Mergenhagen et al. (14)	NR (overall study all sources 8.1%)	59.8% (81,375/136,078)
Acquisto et al. (26)	44.8% (52/116)	57.6% (30/52)
Hitchcock et al. (35)	18.3% (55/300)	63.6% (35/55)
Gunderson et al. (24)	34.7% (58/167)	62.1% (36/58)
Petry et al. (36)	14.6% (80/548)	65% (52/80)

### What would the laboratory, or the FDA, say about the validation data for non-pneumonia off-label use?

As mentioned, off-label use of an FDA-cleared test requires validation as an LDT. Depending on the clinical scenario, low sensitivity during validation may be acceptable under the caveat that testing cannot be used to rule out disease. In the case of MRSA nares colonization screening inference, it is being used to predict or rule out the possibility of MRSA disease and remove or stop initiation of anti-MRSA therapy. Let us compare two FDA-cleared tests with indications for use that include “detection of MRSA” from the relevant site of infection. The BioFire pneumonia panel clinical trial showed a sensitivity of 93.7%, and the Cepheid Xpert MRSA SSTI showed a sensitivity of 93.8% compared to culture for the detection of MRSA in lower respiratory and wound swab samples, respectively. The respective sensitivities of these assays give high confidence that negative results are reliable for rule out. As we have shown, the sensitivity of an MRSA nares colonization screening test can predict true MRSA SSTI disease <65% of the time. As an LDT, it would be unlikely that any laboratory would accept a sensitivity of <65% during validation for MRSA “rule out” via nares colonization screening. It would also be unlikely that the FDA would approve a sensitivity or positive percent agreement <65% for this purpose either.

### Conclusion

In summary, inference of MRSA infection in non-pneumonia syndromes from MRSA nares colonization screening is largely unhelpful due to pre-test probability of low colonization and infection rates, which had declined in recent years (37, 38). Negative test results provide a false sense of “safety to de-escalate” since nearly half or more of true positive cases will test negative. Instead of using a test off-label without adequate validation, diagnostic stewardship efforts should be made to bring in a test that is FDA cleared for this indication or develop a similar LDT where appropriate, using material from the site of infection when possible, rather than nasal colonization as a surrogate (39). In addition, restricting unnecessary vancomycin use in non-pneumonia syndromes seems like an area ripe for clinician decision support tools based on electronic health record data, such as prior MRSA infections or colonization and recent antibiotic exposures. (39).

**Erin H. Graf and Jennifer M. Bosquez**
